# The PCV3 Cap Virus-like Particle Vaccine with the Chimeric PCV2-Neutralizing Epitope Gene Is Effective in Mice

**DOI:** 10.3390/vetsci11060264

**Published:** 2024-06-08

**Authors:** Xingchen Wu, Qikai Wang, Wang Lu, Ying Wang, Zehao Han, Libin Liang, Shimin Gao, Haili Ma, Xiaomao Luo

**Affiliations:** 1 College of Veterinary Medicine, Shanxi Agricultural University, Jinzhong 030801, China; wuxingchen@sxau.edu.cn (X.W.);; 2 Shanxi Academy of Advanced Research and Innovation, Taiyuan 030012, China

**Keywords:** porcine circovirus type 3, capsid, neutralizing epitope, virus-like particle vaccine, immunogenicity

## Abstract

**Simple Summary:**

Porcine circovirus type 3 (PCV3) infection can lead to clinical symptoms in weaned piglets similar to porcine circovirus type 2 (PCV2) infection. The increasing incidence of PCV2 and PCV3 infections, along with the prevalence of coinfections, has led to significant economic losses in the swine industry. Currently, there are no commercial vaccines available for PCV3, and PCV2 vaccines do not provide cross-protection against PCV3. This study optimized the *cap3* sequence based on the codon preferences of *E. coli* and mammalian cells, resulting in the production of highly immunogenic PCV3 virus-like particles (VLPs). Animal experiments with various immunization strategies showed that pCap3-Cap2E VLPs significantly enhanced both humoral and cellular immune responses. This improvement not only reduced the PCV3 viral load in the lungs but also alleviated lung damage. These findings offer valuable insights into a potential strategy for the efficient development of a PCV3 vaccine.

**Abstract:**

Porcine circovirus type 3 (PCV3) infection can cause symptoms similar to those of porcine circovirus type 2 (PCV2) infection, and coinfections with both PCV2 and PCV3 are observed in the swine industry. Consequently, developing chimeric vaccines is essential to prevent and control porcine circovirus infections. In this study, we used both *E. coli* and mammalian expression systems to express PCV3 Cap (Cap3) and a chimeric gene containing the PCV2-neutralizing epitope within the PCV3 Cap (Cap3-Cap2E), which were assembled into virus-like particle (VLP) vaccines. We found that Cap3 lacking nuclear localization signal (NLS) could not form VLPs, while Cap3 with a His-tag successfully assembled into VLPs. Additionally, the chimeric of PCV2-neutralizing epitopes did not interfere with the assembly process of VLPs. Various immunization approaches revealed that pCap3-Cap2E VLP vaccines were capable of activating high PCV3 Cap-specific antibody levels and effectively neutralizing both PCV3 and PCV2. Furthermore, pCap3-Cap2E VLPs demonstrated a potent ability to activate cellular immunity, protecting against PCV3 infection and preventing lung damage in mice. In conclusion, this study successfully developed a PCV3 Cap VLP vaccine incorporating chimeric PCV2-neutralizing epitope genes, providing new perspectives for PCV3 vaccine development.

## 1. Introduction

Porcine circovirus (PCV) is classified within the *Circovirus* family and genus. Currently, there are four types of PCVs identified as PCV1, PCV2, PCV3, and PCV4 [1]. PCV2 infection is known to result in immune suppression, diminishing resistance to various pathogens and causing secondary infection [2]. Previous studies have indicated that coinfections with PCV2 and PCV3 are prevalent in pig farms [3]. PCV3 infection has been associated with clinical symptoms similar to those observed in porcine dermatitis and nephropathy syndrome [4]. PCV3 detected in the colostrum of sows highlighted its potential for early transmission [5]. However, there is no commercialized vaccine for PCV3, which induces significant economic losses to the swine industry [6].

The genome of PCV3 is approximately 2000 bp and consists of single-stranded circular DNA. Genetic evolutionary analyses reveal that PCV3 shares approximately 37% homologous sequence with PCV2 and PCV1. PCV3 is mainly composed of three open reading frames (ORFs), ORF1 and ORF2 coding replicase (Rep) and capsid (Cap) proteins. The role of ORF3 in PCV3 remains unclear. Although the homology of amino acids between PCV3 Rep and PCV2 Rep is approximately 48%, the homology of the Cap is as low as approximately 26% [7,8]. The Cap is crucial for the antigenic properties of PCV2 and PCV3 and plays a pivotal role in activating immune response. The antigenic epitopes of PCV2 Cap (Cap2), including neutralizing epitopes, are key to understanding and developing effective vaccines against PCV2 infection [9]. PCV3 Cap3 (Cap3) is characterized by two significant amino acid mutations (A24V and R27K). These mutations divide PCV3 into three primary lineages as follows: PCV3a (24A and 27R), PCV3b (24A and 27K), and PCV3c (24V and 27K) [10]. The abundance of arginine at the beginning of the Cap3 sequence blocks the expression of Cap3 in *Escherichia coli* (*E. coli*), increasing challenges for research and vaccine development [11]. To express some Cap3 variants, the nuclear localization signal (NLS) peptides were generally truncated. However, the effect of Cap3 NLS on the formation of virus-like particles (VLPs) remains to be further investigated, as it may impact the effectiveness and viability of VLP vaccines.

VLP represents an ideal model for antigen design in developing subunit vaccines. Compared with live-attenuated vaccines and inactivated vaccines, VLP vaccines are safe because they lack the viral genome required to replicate [12]. As a kind of 20–200 nm particle, lower doses of VLPs can also activate B-cells and T-cells by presenting their encoded T-helper cell epitopes to T-helper cells, enhancing cytokine production and adaptive immune system [13]. Additionally, VLP vaccines show good stability during storage and transportation [14]. Furthermore, VLPs can be engineered with chimeric structures to increase exogenous genes and improve the spectrum of immunity. This advantage also extends to VLPs as specific nanocarriers for antigen delivery, enhancing the potential for targeted vaccine strategies and other therapeutic applications [15].

To ensure the formation of VLPs, an efficient expression system is crucial. The primary systems include the *E. coli* expression system, baculovirus expression system, yeast expression system, and mammalian expression system [16,17,18]. As a common expression system, the *E. coli* expression system shows capabilities of rapid production and high expression. However, it cannot perform post-translational modifications of eukaryotic proteins. Since Cap2 lacks envelope and glycosylation sites, Cap2 can be expressed in the *E. coli* system and self-assembled into VLPs [19]. On the other hand, the mammalian expression system offers the advantage of facilitating glycosylation, and it is capable of expressing chimeric VLPs and VLPs with multiple structural proteins [20]. Despite higher costs, the mammalian system is important for producing candidate vaccines and exploring molecular mechanisms.

In the present study, we used prokaryotic and mammalian expression systems to produce the Cap3 protein, with variations including the presence or absence of His-tag and the optimization of the NLS. This approach was designed to investigate the effects of His-tag and Cap NLS regions on the assembly of VLPs. Additionally, we inserted two neutralizing epitope genes from Cap2 into the C-terminus of Cap3 to assess the effect of incorporating exogenous sequences on VLP assembly. Through the evaluation of various individual and combined immunization strategies using DNA and VLP vaccines, we aimed to identify the most effective immunization regimens. These findings are expected to contribute to the development of innovative VLP-based vaccines targeting PCV2 and PCV3, thereby enhancing our capacity to prevent and control infections caused by these viruses.

## 2. Materials and Methods

### 2.1. Viruses and Cells

The genomic sequences of isolated PCV2 and PCV3 strains were uploaded to the National Center for Biotechnology Information (NCBI) database (GenBank accession numbers PP566975 and PP566974). These strains were stored in the Microbiology and Immunology Laboratory of Shanxi Agricultural University and propagated in PK-15 cells. The viral copies were measured by quantitative polymerase chain reaction (PCR). The human embryonic kidney 293F cell line and porcine kidney 15 cell line were stocked in our lab. HEK-293F were cultured in SMM 293-TII medium (Sino Biological, Beijing, China), and PK-15 cells were cultured in high-glucose Dulbecco’s modified essential medium (Invitrogen, Carlsbad, CA, USA) supplemented with 10% heat-inactivated fetal bovine serum (Tianhang Biotechnology, Huzhou, ZJ, China), 100 U/mL penicillin, and 0.1 mg/mL streptomycin. All cells were used at the exponential phase of growth.

### 2.2. Plasmid Construction

*cap3* was optimized based on the preference of *E. coli* codons, as shown in Figure S1A. The optimized *cap3* sequence was fused with His-tag and inserted into the pET-28a vector (GENEWIZ Biotechnology, Suzhou, JS, China) to produce the recombinant plasmid pET-28a-Cap3 + His. A sequence lacking the optimized NLS at the front end of Cap3 (Cap3△N) resulted in the construction of the recombinant plasmid pET-28a-Cap3△N + His. Furthermore, the optimized *cap3* sequence was synthesized and cloned into the pET-28a vector to generate the recombinant plasmid pET-28a-Cap3. The optimized *Cap3* sequence was also linked to two neutralizing epitopes of *cap2*, named 5′-CACGTGGTCGGTACGCATTT-3′ and 5′-CTGAAAGATCCCCGCTGAATCCA-3′, using a linker (GGGGS). These constructs were inserted into the pET-28a vector to obtain the recombinant plasmid pET-28a-Cap3-Cap2E. For mammalian expression, *cap3* was based on the preference of the codon, as shown in Figure S1B. The optimized *cap3* sequence, the Kozak sequence (GCCACC), and a secreted peptide (MHSSALLCCLVLLTGVRA) were combined with His-tag and then inserted into the pCAGGS vector to obtain the recombinant plasmid pCAGGS-Cap3 + His. Similarly, pCAGGS-Cap3△N + His, pCAGGS-Cap3, and pCAGGS-Cap3-Cap2E were constructed. All primers, designed sequences, and peptides were obtained from Sangon Biotech (Shanghai, China).

### 2.3. Protein Expression and Purification

The recombinant plasmids pET-28a-Cap3 + His, pET-28a-Cap3△N + His, pET-28a-Cap3, and pET-28a Cap3-Cap2E were expressed in the Rosetta strain, producing pCap3 + His, pCap3△N + His, pCap3, and pCap3-Cap2E, respectively. Similarly, PCAGGS-Cap3 + His, pCAGGS-Cap3△N + His, pCAGGS-Cap3, and pCAGGS-Cap3-Cap2E were transfected into HEK-293F cells, and the proteins eCap3 + His, eCap3△N + His, eCap3, and eCap3-Cap2E were obtained. The proteins of pCap3 + His and pCap3△N + His were purified with a His-tag protein purification kit (Beyotime Biotechnology, Shanghai, China) according to the manufactural protocol, which is bound to Ni-NTA resin. However, pCap3, pCap3-Cap2E, eCap3 + His, eCap3△N + His, eCap3, and eCap3-Cap2E were purified by gel filtration chromatography on a HiLoad 16/600 Superdex 200 pg column (Cytiva, Washington, DC, USA) in a buffer containing 20 mM Tris (pH 8.0) and 150 mM NaCl using molecular sieves.

### 2.4. Assembly and Identification of VLPs In Vitro

The purified protein was added to a dialysis bag and placed in an assembly buffer with a 50-fold volume of protein. Briefly, the assembly buffer was composed of 100 mmol/L NaH_2_PO_4_, 0.5 mol/L NaCl, 100 mmol/L KCl, 10 mmol/L Tris-HCl, 0.5% Triton X-100, and 0.1 mmol/L PMSF. The mixture was gently mixed at 4 °C for 48 h, and the buffer was changed three times during this period. After dialysis, a 20 μL sample was applied to a copper mesh and kept for 10 min. Then, the sample was stained with a drop of 2% phosphor-tungstic acid for 90 s and absorbed the dye solution by filter paper. Transmission electron microscopy (TEM) (JEOL, Tokyo, Japan) was used to observe the formation of VLPs after drying.

### 2.5. Animal Experimental Design

Seven-week-old BALB/c mice were randomly divided into 9 groups, each consisting of 18 mice. One group served as the control (Mock), while the remaining 8 groups were immunized with VLPs (100 μg), DNA (100 μg), or a combination of VLPs (50 μg) and DNA (50 μg) via intramuscular injections. The immunizations were performed at 0 d and 14 d. The levels of Cap-specific antibodies were monitored every 7 days following vaccination. Serum samples were collected at 28 d and 35 d post-vaccination for measuring virus-neutralizing antibody levels. At 28 d and 35 d post-vaccination, 6 mice in each group were euthanized to conduct splenic lymphocyte proliferation assay and enzyme-linked immunosorbent assay (ELISA) for detecting IL-4 and IFN-γ. Thirty-five days after vaccination, all mice, except those in the Mock group (intraperitoneal injection with DMEM medium), were challenged by intraperitoneal injection of 1 × 10^5^ copies of PCV3. At 63 days post-vaccination, the mice were euthanized to harvest samples.

### 2.6. Enzyme-Linked Immunosorbent Assay

The peripheral blood plasmas of mice were harvested from randomly six mice in each group at 7 d, 14 d, 21 d, 28 d, and 35 d post-vaccination. The serums were used to measure the levels of PCV3-specific antibodies according to the manufacturer’s instructions for the PCV3 antibody detection kit (Sennoside Biotechnology, Chuzhou, AH, China). The optical density at 450 nm of these samples was detected using a microplate reader. For the detection of IL-4 and IFN-γ, cell supernatants were measured according to the manufacturer’s guidelines using commercial ELISA kits (MEIMIAN, Yancheng, JS, China) [21].

### 2.7. Virus Neutralization Test

The serum was continuously diluted with the culture medium starting from a 1:2 ratio, and then 50 μL diluted serum mixed with 50 μL 200 TCID_50_/100 μL PCV2 or PCV3 were incubated at 37 °C for 1 h. The mixture was added to a 96-well plate containing PK-15 cells for infection. After 72 h, the cells were incubated with rabbit anti-Cap serum at 4 °C and labeled with FITC-conjugated goat anti-rabbit IgG (Solarbio, Beijing, China). The viral loads within these cells were detected, and the titers of PCV2- and PCV3-neutralizing antibodies were calculated based on inhibiting viral infection [22].

### 2.8. Spleen Lymphocyte Proliferation Assay

According to the manufacturer’s instructions, spleen lymphocytes were isolated using a lymphocyte isolation medium (Solarbio, Beijing, China). The splenic lymphocytes were seeded into a 96-well plate and stimulated with 1 MOI PCV3, 5 μg/mL Concanavalin A (ConA) or culture medium for 48 h. Subsequently, 20 μL of CCK-8 solution was added to each well under dark conditions and the mixture was incubated for 2 h. The absorbance was measured at 450 nm. The result was calculated using the following formula: (OD value of PCV3-stimulated well-OD of the blank well)/(OD of negative control well-OD of the blank well).

### 2.9. Statistical Analysis

All statistical analyses were performed using GraphPad Prism 9 (GraphPad Inc., San Diego, CA, USA, 2020). The data are presented as means ± SDs. Comparisons between 2 groups were performed by unpaired Student’s test. Statistical significance was defined as *p* < 0.05.

## 3. Results

### 3.1. Plasmid Construction and Protein Purification

**Figure 1:**
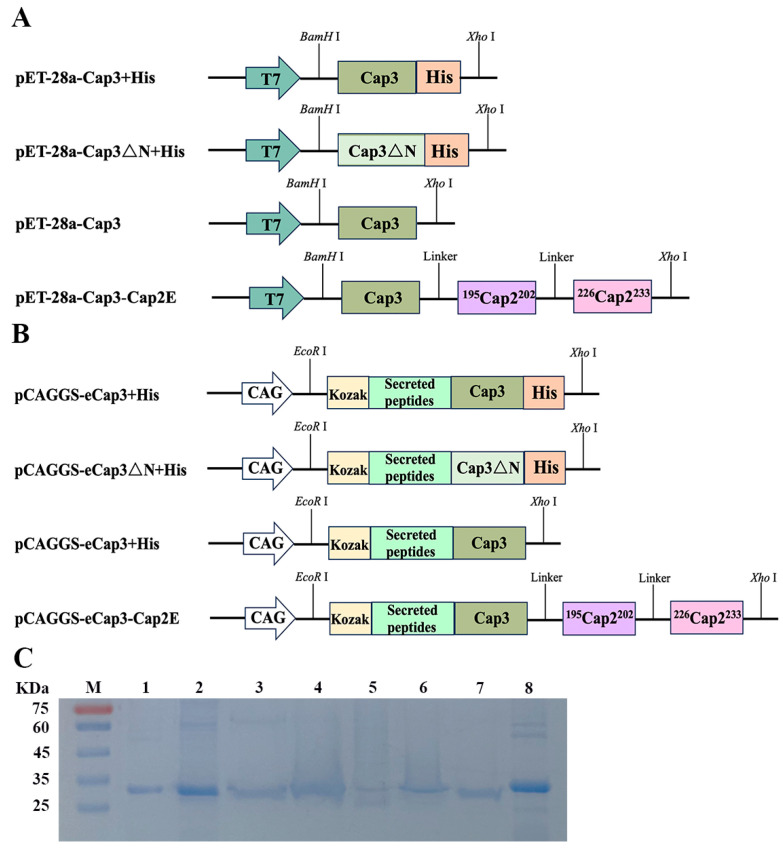
(**A**,**B**) Schematic diagram of expression vector construction. T7 and CAG represent the promoters in the vectors, while Cap3ΔN represents the Cap3 sequence lacking an NLS. The sequences ^195^Cap2^202^ and ^226^Cap2^233^ represent two neutralizing epitopes of Cap2. (**C**) Results of SDS–PAGE following purified proteins. M: protein marker; 1: pCap3 + His; 2: pCap3△N + His; 3: pCap3; 4: pCap3-Cap2E; 5: eCap3 + His; 6: eCap3ΔN + His; 7: eCap3; 8: eCap3-Cap2E.

To effectively prevent infections by PCV2, PCV3, and coinfections, we constructed recombinant plasmids for completing prokaryotic and eukaryotic expression. Based on the codon preference of *E. coli*, we optimized the codon for PCV3 Cap (*cap3*), resulting in the expression of pET-28a-Cap3 + His (Figure S1A and Figure 1A). Otherwise, we constructed the pET-28a-Cap3∆N + His plasmid, which lacks an NLS (Figure 1A). By optimizing the *cap3* codon relative to mammalian codon preferences and adding the Kozak and secretory peptide sequences to the 5′ end of Cap3, we engineered the pCAGGS-Cap3 + His and pCAGGS-Cap3ΔN + His vectors (Figure S1B and Figure 1B). However, proteins expressed with the pCAGGS-Cap3 + His and pCAGGS-Cap3ΔN + His vectors could not be purified via affinity chromatography. Therefore, recombinant plasmids of pET-28a-Cap3 and pCAGGS-Cap3 without His-tag were developed. Additionally, we constructed the recombinant plasmids pET-28a-Cap3-Cap2E and pCAGGS-Cap3-Cap2E by designing two distinct Cap2-neutralizing epitope genes to insert the 3′ end of Cap3 (Figure 1A,B). The proteins expressed by pET-28a-Cap3 + His, pET-28a-Cap3△N + His, pET-28a-Cap3, pET-28a-Cap3-Cap2E, pCAGGS-Cap3 + His, pCAGGS-Cap3△N + His, pCAGGS-Cap3, and pCAGGS-Cap3-Cap2E are represented by pCap3 + His, pCap3△N + His, pCap3, pCap3-Cap2E, eCap3 + His, eCap3△N + His, eCap3, and eCap3-Cap2E, respectively. Importantly, pCap3 + His and pCap3△N + His were purified through affinity chromatography, while pCap3, pCap3-Cap2E, eCap3 + His, eCap3ΔN + His, eCap3, and eCap3-Cap2E were processed using molecular sieves (Figure S2). The purified proteins were analyzed by SDS-PAGE and observed as distinct bands at approximately 26~30 kDa (Figure 1C).

### 3.2. The Absence of an NLS Prevented the Formation of PCV3 Cap VLPs

**Figure 2:**
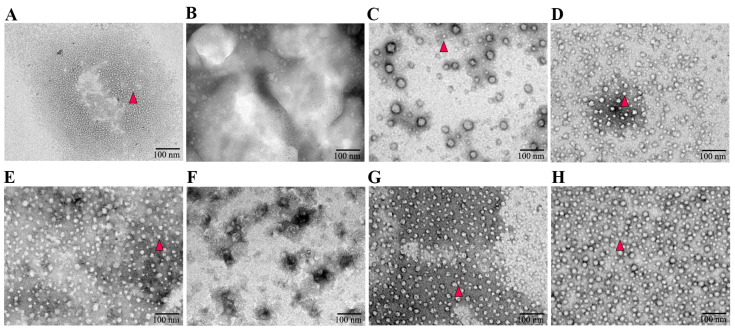
Negatively stained TEM images of the assembled VLPs. (**A**–**H**) represent the VLPs of pCap3 + His, pCap3△N + His, pCap3, pCap3-Cap2E, eCap3 + His, eCap3△N + His, eCap3, and eCap3-Cap2E. The magnification of TEM images is 50,000 times, and the scale is 100 nm. The formed VLPs are highlighted with red triangles.

To verify the characterization of the assembled VLPs, TEM was employed to examine products following purified protein dialysis in the assembly buffer. The recombinant proteins pCap3 + His, pCap3, eCap3 + His, and eCap3 were successfully assembled into VLPs with an approximate diameter of 17 nm. pCap3-Cap2E and eCap3-Cap2E formed VLPs with a slightly larger diameter, approximately 20 nm. However, the variants pCap3∆N + His and eCap3∆N + His were incapable of forming VLPs, emphasizing the critical role of NLS in VLP assembly (Figure 2).

### 3.3. pCap3-Cap2E VLPs Activated Higher Humoral Immune Responses Than the Other Inoculated Groups

**Figure 3:**
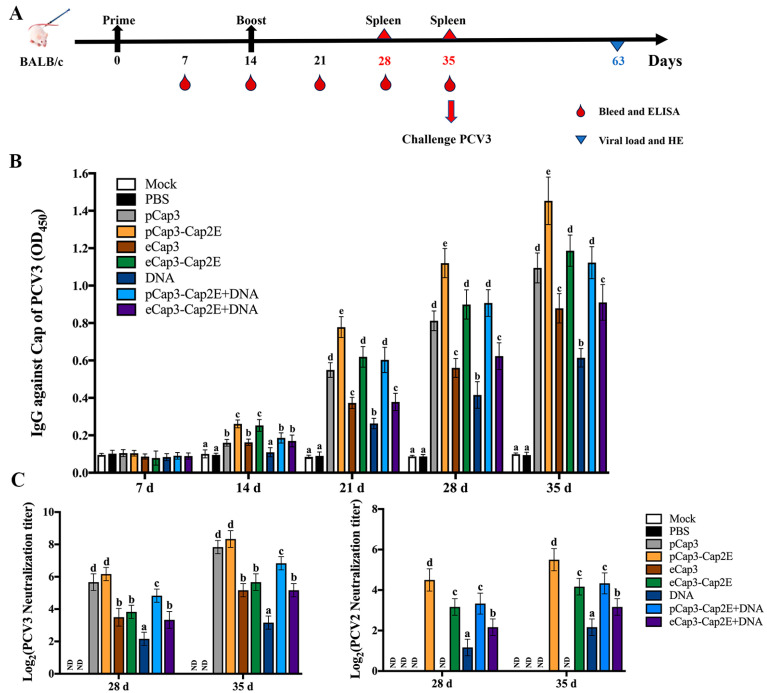
(**A**) Inoculation schedule and blood collection, spleen collection, and challenge times. (**B**) Serum samples from vaccinated mice were collected to measure PCV3 Cap-specific antibody levels at the indicated time points by ELISA. (**C**) PCV2 and PCV3 were incubated with serum from 28 d and 35 d post-inoculated mice and then stimulated with PK-15 cells for 72 h. PCV2- and PCV3-neutralizing antibody levels were calculated. ND indicates that no detection was found. Identical lowercase letters (a–e) across the results suggest no significant differences among groups at the same time point, whereas different lowercase letters suggest significant differences among groups at the indicated time point in (**B**,**C**).

To detect the ability of VLP vaccines to stimulate humoral immunity, we measured serum antibodies at 7 d intervals after the mice were vaccinated at 0 d and 14 d with the various groups of VLPs and the eukaryotic expression plasmid pCAGGS-Cap3-Cap2E (DNA) (Figure 3A). The results for Cap-specific antibodies indicated that anti-PCV3 antibody levels in the inoculated mice progressively increased following booster immunizations. In particular, at 21 d, 28 d, and 35 d post-inoculation, the anti-PCV3 antibody levels in the pCap3, pCap3-Cap2E, eCap3-Cap2E, and pCap3-Cap2E + DNA groups were significantly upregulated compared with those in the eCap3, DNA, and eCap3-Cap2E + DNA groups. Notably, the anti-PCV3 antibody concentration in the pCap3-Cap2E group was significantly higher than that in the pCap3, eCap3-Cap2E, and pCap3-Cap2E + DNA groups (Figure 3B). The levels of PCV3-neutralizing antibodies were markedly higher in the pCap3 and pCap3-Cap2E groups than in the other vaccine groups. No PCV2-neutralizing antibodies were detected in the pCap3 and eCap3 groups. However, the levels were significantly increased in the pCap3-Cap2E group compared with those in the eCap3-Cap2E, DNA, pCap3-Cap2E + DNA, and eCap3-Cap2E + DNA groups (Figure 3C). These findings indicate that pCap3-Cap2E VLPs can induce higher humoral immune responses in mice and effectively neutralize both PCV2 and PCV3.

### 3.4. pCap3-Cap2E VLPs Activated Higher Cellular Immune Responses Than the Other Inoculation Groups

**Figure 4:**
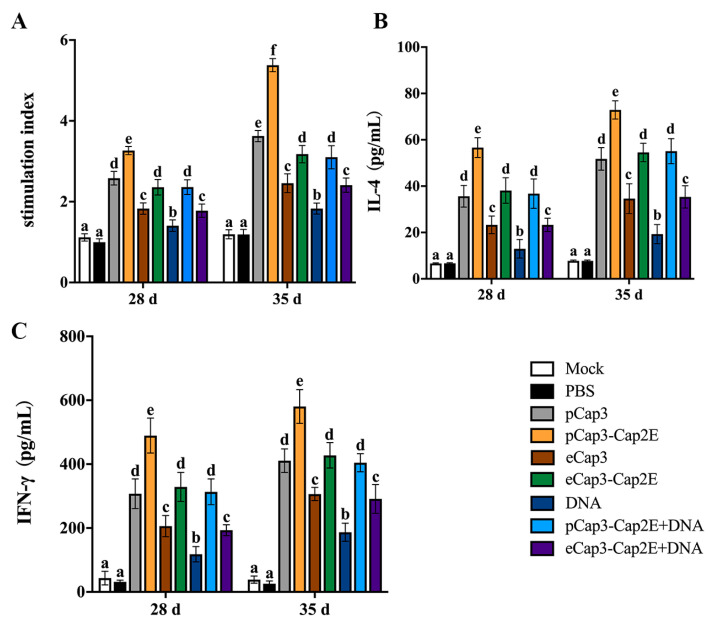
At 28 d and 35 d post-vaccination, spleen lymphocytes were isolated and seeded onto 96-well plates. (**A**) These cells were then stimulated with PCV3 for 48 h, using ConA as a positive control and culture medium as a negative control, to assess the proliferation of spleen lymphocytes by the CCK-8 assay. (**B**,**C**) The levels of IL-4 and IFN-γ in the supernatant of lymphocytes from different groups post-PCV3 stimulation were measured using ELISA kits. Identical lowercase letters (a–f) across the results suggest no significant differences among groups at the same time point, whereas different lowercase letters suggest significant differences among groups at the indicated time point in (**B**,**C**).

To further examine the cellular immune response level in mice, splenic lymphocytes were harvested at 28 d and 35 d post-inoculation to detect proliferative activity. The results revealed that lymphocyte proliferation in the pCap3, pCap3-Cap2E, eCap3-Cap2E, and pCap3-Cap2E + DNA groups were significantly upregulated compared with that in the eCap3, DNA, and eCap3-Cap2E + DNA groups. Moreover, proliferation was significantly higher in the pCap3-Cap2E group compared with that in the pCap3, eCap3-Cap2E, and pCap3-Cap2E + DNA groups (Figure 4A). Furthermore, the levels of IL-4 and IFN-γ in mouse spleen lymphocytes significantly increased in the pCap3, pCap3-Cap2E, eCap3-Cap2E, and pCap3-Cap2E + DNA groups compared with those in the eCap3, DNA, and eCap3-Cap2E + DNA groups. Additionally, IL-4 and IFN-γ production in the pCap3-Cap2E group was markedly higher compared with that in the pCap3, eCap3-Cap2E, and pCap3-Cap2E + DNA groups (Figure 4B,C). These results indicate that pCap3-Cap2E VLPs can enhance the cellular immune responses in mice.

### 3.5. Viral Load Was Lower and Pathological Damage to the Lungs Was Less Severe in Mice Vaccinated with pCap3-Cap2E VLPs Than in Other Groups

**Figure 5:**
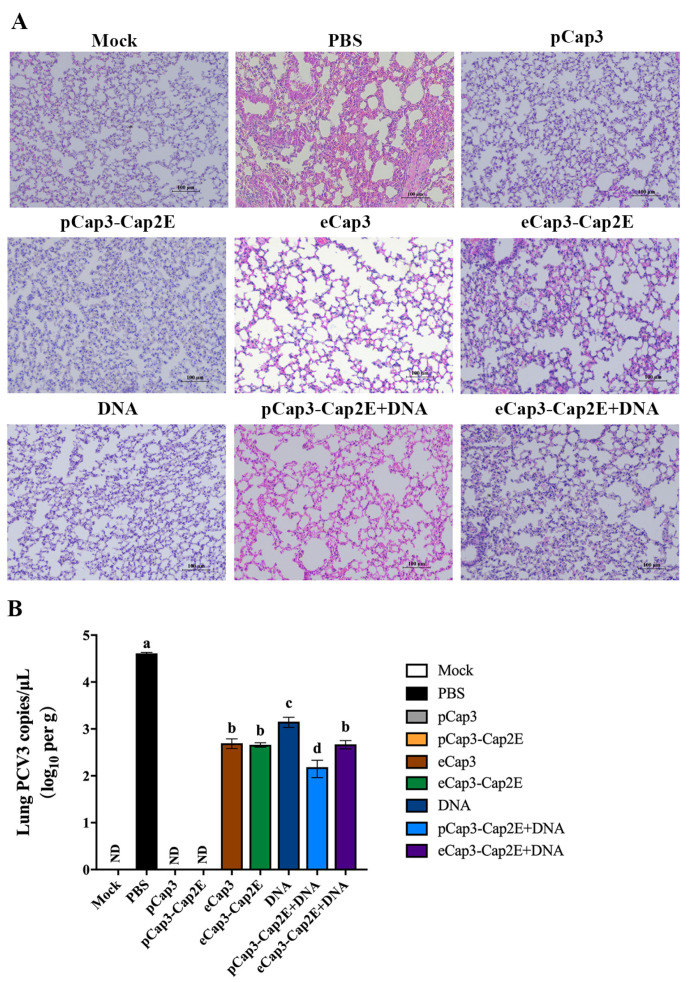
(**A**) Mice from each group were sacrificed, and lung tissue was harvested at 63 days post-vaccination. The lungs were processed into paraffin-embedded tissue sections, which were then stained with HE to observe pathological changes under a microscope. The scale bar represents 100 μm. (**B**) The copies of PCV3 in the lungs of mice from each group were quantified using qPCR. ND indicates that no detection was found. Identical lowercase letters (a–d) across the results suggest no significant differences among groups at the same time point, whereas different lowercase letters suggest significant differences among groups at the indicated time point.

To detect the protective efficacy of vaccines against PCV3 infection, we analyzed lung tissue pathology after the PCV3 challenged for 28 d. The results showed significant lesions characterized by inflammatory cell infiltration, alveolar wall thickening, and damage of alveolar epithelial cells in the lungs of mice in the PBS group (Figure 5A). Mice in the eCap3, eCap3-Cap2E, DNA, pCap3-Cap2E + DNA, and eCap3-Cap2E + DNA groups also exhibited moderate lung lesions, although these lesions were notably less severe than those observed in the PBS group (Figure 5A). The lesions were not shown in the lungs of mice in the pCap3 and pCap3-Cap2E groups (Figure 5A). The viral load in the lungs was quantified using qPCR at 28 d post-infection. The results indicated that the viral loads in the pCap3, pCap3-Cap2E, eCap3-Cap2E, and pCap3-Cap2E + DNA groups were significantly lower compared with those in the eCap3, DNA, and eCap3-Cap2E + DNA groups. Furthermore, the viral load in the pCap3-Cap2E group was notably lower than those in the pCap3, eCap3-Cap2E, and pCap3-Cap2E + DNA groups (Figure 5B). These results demonstrate that pCap3–Cap2E VLPs can reduce viral presence and attenuate lung lessons following the PCV3 challenge.

## 4. Discussion

PCV2 and PCV3 have emerged as prevalent pathogens worldwide, with a notably high incidence of coinfection, which resulted in huge economic losses in the swine industry recently [23,24]. Both PCV2 and PCV3 infections exhibit similar clinical symptoms, increasing difficulties in diagnosis and prevention [25,26]. Vaccination represents the most effective strategy for the control and prevention of infections caused by PCV2 and PCV3. Previous research has proven that Cap2 and Cap3 can self-assemble into VLPs in vitro. VLPs show stronger immunogenicity than other subunit vaccines and can induce higher humoral and cellular immune responses. Therefore, VLPs represent an optimal antigen model to develop subunit vaccines against PCV2 and PCV3 [12,15,27]. The present study investigated the preparation and immunogenic evaluation of PCV3 VLPs that incorporate PCV2-neutralizing epitope genes, aiming to lay a scientific foundation for the development of novel VLP-based vaccines against PCV2 and PCV3 infections, including coinfections.

Several PCV2 vaccines were designed with truncated Caps lacking 1-41 amino acid NLS peptides, highlighting the essential role of NLS peptides in the assembly of PCV2 particles and the potential contribution to immune responses [28,29,30]. The NLS located at the front end of Cap3 contains an arginine-rich sequence for targeting the nucleus. However, this NLS can hinder the expression of the Cap3 protein in vitro [6,11]. To explore the effects of NLS absence on VLP formation, this study employed both *E. coli* and mammalian expression systems to express Cap3 and a variant of Cap3△N lacking an NLS, respectively. These results demonstrated that Cap3 with His-tag could assemble into VLPs approximately 17 nm in diameter. In contrast, the Cap3△N + His variant failed to assemble into VLPs, underscoring the significance of NLS in the process of VLPs formation.

Secretion signal peptides are sequence motifs regulating proteins to secrete into the extracellular via the endoplasmic reticulum Golgi [31]. In this study, a mammalian expression system in which a signal peptide was incorporated at the N-terminus of Cap3 to facilitate its secretion from 293F cells was used to express the Cap protein. However, it was observed that the recombinant Cap3 almost did not secret into the extracellular space, instead being expressed intracellularly, as expected. The low level of recombinant protein presented significant challenges for protein purification and VLP assembly. Additionally, the high costs associated with eukaryotic expression systems pose additional hurdles to the production of VLPs, underscoring the necessity for optimized strategies to enhance protein secretion and expression efficiency.

His-tag is the most commonly used tag for protein detection and purification [32]. Although many subunit vaccines contain purification tags, such as His or Sumo-tag, these tags may interfere with the assembly of VLPs [33,34]. In this study, we investigated how the presence or absence of tags at the C-terminus of Cap3 influences VLP assembly efficiency. Using the *E. coli* expression system, we expressed Cap3 + His and purified the protein via affinity chromatography. The results indicated that the His-tag did not impair the assembly of Cap3 + His VLPs. However, Cap3 + His could not form VLPs when Cap3 + His was expressed using the mammalian expression system, as affinity chromatography was ineffective for purification, using gel filtration chromatography instead.

To effectively prevent PCV2 and PCV3 infections, two neutralizing epitope genes from Cap2 were inserted into the C-terminus of Cap3. The results showed that the recombinant Cap3-Cap2E protein was successfully assembled into VLPs. This finding demonstrated that the indicated neutralizing epitope genes from Cap2 did not affect the assembly of VLPs. Previous studies explored the assembly of VLPs by replacing the stem and TM regions of the dengue fever virus E protein with those of the vesicular stomatitis virus G protein, which were slightly larger than those assembled from the wild-type components [35,36]. Notably, the diameter of VLPs produced by the recombinant Cap3-Cap2E protein, which contains the neutralizing epitope of *cap2*, was approximately 20 nm. This size is larger than that of the VLPs assembled from the recombinant Cap3 + His and Cap3 proteins.

Based on current progress in defining cross-protective immunity, the co-immunization of multiple vaccines can significantly impact protection efficacy [37]. Co-immunization with DNA and VLP efficiently induced better protective immunity against chikungunya virus infection [38]. To assess immune responses, mice were immunized with different protocols, followed by immunological detection and PCV3 challenge. This study showed that the levels of specific antibodies, splenic lymphocyte proliferation, neutralizing antibodies, and the cytokines IL-4 and IFN-γ were significantly activated in the VLP, DNA, and VLP + DNA groups compared with those in the control group at 35 d post-immunization. Notably, the immune response in the group receiving pCap3-Cap2E was higher than that in all the other immunized groups. This group also exhibited a substantial reduction in viral load within the lungs relative to that of the controls. Previous research proved that a new PCV2 DNA vaccine expressing the gC1qR binding site mutant Cap induced stronger humoral and cellular immune responses than a PCV2 DNA vaccine expressing the wild-type Cap2 and protected mice from PCV2 infection and lung lesions [28]. The recombinant baculoviruses expressing the Rep-Cap fusion protein of PCV2 developed higher PCV2-specific neutralizing antibody titers and enhanced protective efficacy in mice [39]. Examination of lung pathology revealed that there was no significant bleeding, proliferation, inflammatory cell infiltration, or other lesions in the pCap3-Cap2E group, indicating a protective effect. The Cap2-neutralizing epitope inserted into pCap3-Cap2E serves as an immunodominant determinant, suggesting that the chimeric-neutralizing epitopes of pCap3-Cap2E VLPs are capable of enhancing broad immune responses, highlighting their potential effectiveness as vaccine components against PCV2 and PCV3 infections. Purified hepatitis E virus (HEV)-PCV2 chimeric VLPs exhibited optimal antigenicity and immunogenicity [40]. Our results are consistent with these findings on the protective efficacy of VLP vaccines. In the next study, we will further verify the protection of pCap3-Cap2E VLPs in pigs. Meanwhile, the data indicate that incorporating exogenous genes does not impact the assembly of PCV3 VLPs, providing innovative approaches to the development of combination vaccines targeting multiple porcine pathogens.

## 5. Conclusions

In summary, this study presents evidence that the VLP vaccine consisting of chimeric pCap3-Cap2E effectively prevents PCV3 infection in mice by enhancing both cellular immune responses and humoral immunity. These findings offer valuable insights into a potential strategy for the development of a PCV3 vaccine.

## Data Availability

The data that support the findings of this study are available from the corresponding author upon reasonable request.

## Supplementary Materials

The following supporting information can be downloaded at: https://www.mdpi.com/article/10.3390/vetsci11060264/s1, Figure S1: Comparison of sequences after *cap3* optimization; Figure S2: The identification of different purification proteins.
