# Supplementary material for: The PCV3 Cap Virus-like Particle Vaccine with the Chimeric PCV2-Neutralizing Epitope Gene Is Effective in Mice
Source: Vet Sci. 2024 Jun 8;11(6):264. doi: 10.3390/vetsci11060264 (PMC11209062; doi:10.3390/vetsci11060264)
Supplement: Supplementary file 1 [file vetsci-11-00264-s001.zip › vetsci-2976403-supplementary.pdf]

Supplementary Material

The PCV3 Cap Virus-Like Particle Vaccine with the Chimeric PCV2-Neutralizing Epitope Gene Is Effective in Mice

Xingchen Wu [1,†], Qikai Wang [1,†], Wang Lu [1], Ying Wang [1], Zehao Han [1], Libin Liang [1], Shimin Gao [1], Haili Ma [1,*], Xiaomao Luo [1,2*]

*** Correspondence:** vm210028@sxau.edu.cn (H.M.); xmluo@sxau.edu.cn (X.L.)

1 Supplementary Figures

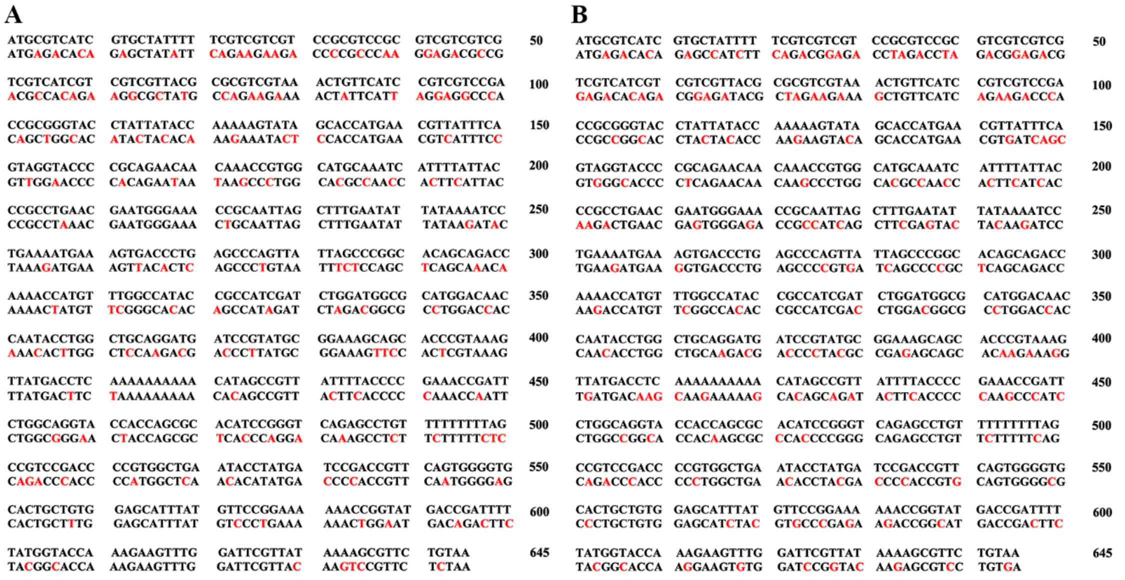

Fig S1. Comparison of sequences after *cap3* optimization. (A) The optimized codons of *cap3* are based on *E. coli* preference. **(B)** The optimized codons of *cap3* are based on Mammalian preference. The optimized codons are marked in red.

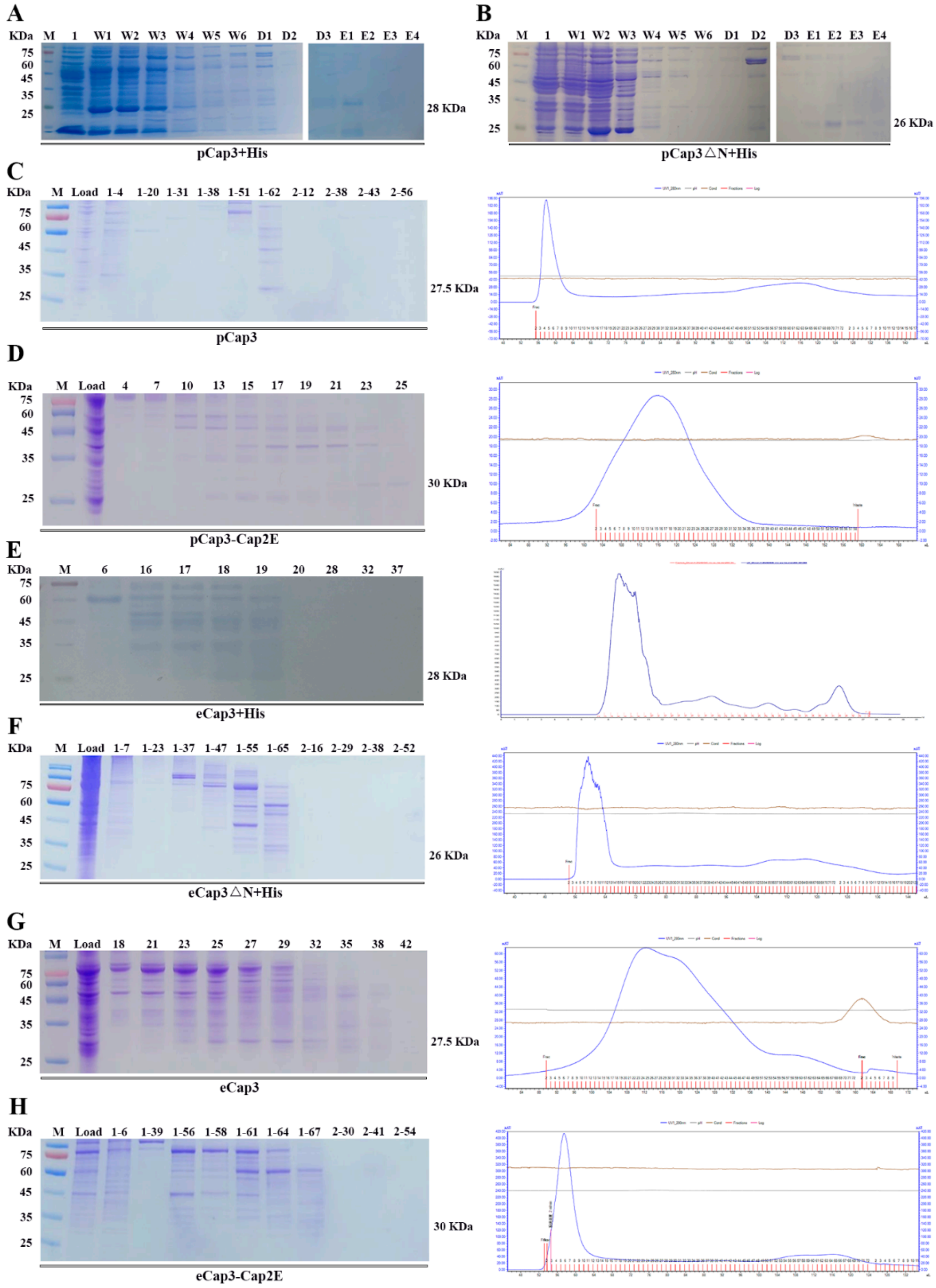

Fig S2. The identification of different purification proteins. (A-B) Results of SDS-PAGE following purification of pCap3 + His and pCap3ΔN + His. M: Protein marker; 1: Flow-through; W1-W5: Wash solution containing 20 mM imidazole; D1-D3: Wash solution containing 80 mM imidazole; E1-E4: Elution containing 250 mM imidazole. (**C-H**) Results of SDS–PAGE following purification of pCap3, pCap3-Cap2E, eCap3 + His, eCap3ΔN + His, eCap3, and eCap3-Cap2E. M: Protein marker; Load: Bacterial lysis (C, D) or cell lysis (E-H) supernatant; Different numbers represent different fractions after chromatography column purification at different periods. The analysis of purification proteins by Gel filtration chromatography (GFC).
